# Impact of urbanization trends on production of key staple crops

**DOI:** 10.1007/s13280-021-01674-z

**Published:** 2021-11-29

**Authors:** José F. Andrade, Kenneth G. Cassman, Juan I. Rattalino Edreira, Fahmuddin Agus, Abdullahi Bala, Nanyan Deng, Patricio Grassini

**Affiliations:** 1 Department of Agronomy and Horticulture, University of Nebraska-Lincoln, Lincoln, NE 68583-0915 USA; 2 Departamento de Producción Vegetal, Cátedra de Cerealicultura, Facultad de Agronomía, Universidad de Buenos Aires, IFEVA-CONICET, Av. San Martin 4453, C1417, Buenos Aires, Argentina; 3 Indonesian Center for Agricultural Land Resources Research and Development, Indonesian Agency for Agricultural Research and Development, Bogor, 16114 Indonesia; 4 Department of Soil Science, Federal University of Technology Minna, P.M.B 65 Gidan-Kwano, Minna, Niger Nigeria; 5 National Key Laboratory of Crop Genetic Improvement, MARA Key Laboratory of Crop Ecophysiology and Farming System in the Middle Reaches of the Yangtze River, College of Plant Science and Technology, Huazhong Agricultural University, Wuhan, 430070 Hubei China

**Keywords:** Land conversion, Maize, Rice, Self-sufficiency, Staple crops, Urbanization, Yield potential

## Abstract

Urbanization has appropriated millions of hectares of cropland, and this trend will persist as cities continue to expand. We estimate the impact of this conversion as the amount of land needed elsewhere to give the same yield potential as determined by differences in climate and soil properties. Robust spatial upscaling techniques, well-validated crop simulation models, and soil, climate, and cropping system databases are employed with a focus on populous countries with high rates of land conversion. We find that converted cropland is 30–40% more productive than new cropland, which means that projection of food production potential must account for expected cropland loss to urbanization. Policies that protect existing farmland from urbanization would help relieve pressure on expansion of agriculture into natural ecosystems.

**Supplementary Information:**

The online version contains supplementary material available at 10.1007/s13280-021-01674-z.

## Introduction

Prior to the establishment of global supply chains and modern transport systems, most large cities were provisioned with food from surrounding farmland. For this reason, many of the world’s major cities were established in locations with good soils and adequate rainfall or irrigation to ensure a dependable food supply (Carter and Dale 1974). Hence, as urban populations grow, surrounding highly productive croplands producing staple food crops are converted to urban uses as well as higher-value vegetable and specialty crops. With rapid urban growth rates of the past 30 years, global conversion of cropland near urban areas has been occurring at 1.2 million ha year^−1^ (van Vliet 2019). Continuing rapid urbanization suggests that conversion of agricultural lands for urban uses will proceed rapidly into the foreseeable future (United Nations 2018). At the same time, land area devoted to production of staple food crops is expanding worldwide at a rapid rate (Cassman and Grassini 2020). Much of this expansion occurs far from cities and comes at the expense of rainforests, wetlands, and grasslands, which reduces biodiversity and water resources supported by these natural habitats (Tilman et al. 2011; Mulyani et al. 2016; Lark et al. 2020).

At issue is the degree to which spatial changes in cropland affect staple crop production capacity and yield stability due to differences in soil and climate. For example, if soil of new cropland holds less water or is located in a region with harsher climate than converted cropland, a decrease in yield potential and yield stability is likely to occur, or vice versa. Here we define yield potential as the yield obtained with good crop and soil management that minimizes losses from abiotic and biotic stresses (Evans 1993). Robust estimates of differences in yield potential between new and converted croplands are needed to assess the impact on current and future production capacity of key staple crops. From a purely economic point of view, reduction in production potential of a given commodity could be covered by imports assuming a country has the purchasing power to do so. However, due to repeated cycles of price spikes and embargoes on grain exports by several major grain exporting countries (Mitra and Josling 2009), populous countries often establish policies to achieve self-sufficiency for specific staple food crops to avoid supply chain disruptions that jeopardize food security (Clapp 2017). Previous studies assessing the impact of changes in cropland due to urbanization have been based on total cropland, without distinguishing by crop type, using calories equivalents to determine average changes in productivity between new and converted land (Supplementary Table S1). In contrast, we are not aware of any study that has investigated how changes in cropland due to urbanization would affect the potential capacity of specific countries to produce key staple crops, such as rice in Asia or maize in Sub-Saharan Africa.

This study utilizes robust spatial upscaling techniques, well-validated crop-specific simulation models, and soil, climate, and cropping system databases at finer spatial resolution, using primary data sources as much as possible, to estimate yield potential and yield stability of current and newly developed croplands (Grassini et al. 2015; van Bussel et al. 2015). To determine the impact on crop production, we compare yield potential and yield stability of cropland in regions with contracting or expanding production area in the first decade of the new millennium for rice in China (irrigated) and Indonesia (rainfed and irrigated) and rainfed maize in Nigeria. These countries and crops were selected because (1) they have large populations and associated food demand, (2) are projected to undergo rapid land-use change due to urbanization (Seto et al. 2012; d’Amour et al. 2017), and (3) the evaluated crops represent major staples in national diets, account for a large proportion of total farmland in each country (http://www.faostat.org), and their domestic demand will increase in the future (van Ittersum et al. 2016; Agus et al. 2019; Deng et al. 2019).

## Materials and methods

### Estimation of land productivity

Our analysis is based on comparison of annual crop yield potential of converted and new croplands rather than on differences in current farm yields of both land categories. The latter approach can mask differences in the inherent productive capacity of agricultural land, as determined by soil quality and climate, due to differences in sophistication of crop and soil management practices or access to inputs and markets, all of which can limit yields (Lobell et al. 2009). In many developing countries, and especially at the frontiers of current agricultural areas, farmers have limited access to inputs, equipment, supporting services and technologies. However, we also evaluated current average yields and the results are presented in Supplementary Table S2 although we believe these results are less useful. For example, substantial funding is allocated by government agencies and charitable foundations (e.g., Bill and Melinda Gates Foundation, CGIAR, USAID-Feed the Future Initiative) to improve farmer access to markets, technologies, and information in developing countries. Therefore, an analysis to inform national policies concerning agricultural development and land-use policies based on current yields would not only mask the potential impact of spatial changes in cropland based on use of modern farming practices, but it would rapidly become outdated as farmers gain access to markets, technologies, and information.

Yield potential is the yield of a crop cultivar when grown with water and nutrients non-limiting and biotic stress effectively controlled (Evans 1993; van Ittersum and Rabbinge 1997). Under these conditions, crop growth rate is determined by solar radiation, temperature, atmospheric CO_2_, and genetic traits that govern the length of growing period and light interception by the crop. For rainfed crops, rainfall amount and distribution and soil water holding capacity also impose an upper limit to crop productivity. Hence, yield potential is the most relevant parameter for estimating crop production potential of irrigated crop systems, while water-limited yield potential is the appropriate benchmark for rainfed crops. Current yield is defined as the yield achieved in farmer’s fields in recent years within a defined spatial unit.

We used crop models to estimate yield potential in each country. The main challenge to obtain accurate simulations using crop models is the availability of high-quality primary data for climate, soil, and crop management, which are the most sensitive parameters determining yield potential. Weather stations are sparse and soil and cropping system information is rarely adequate to estimate yield potential for many crop production areas in developing countries. To overcome this limitation, we used the Global Yield Gap Atlas (http://www.yieldgap.org, GYGA) “bottom-up” spatial framework that identifies the minimum number of sites needed for robust estimation of yield potential at local, regional, and national scales (van Wart et al. 2013; van Bussel et al. 2015; Hochman et al. 2016, Morel et al. 2016; Rattalino Edreira et al. 2021).

The GYGA framework delimits climate zones (CZ) based on spatial variation in three key variables influencing crop growth and yield: growing degree days, temperature seasonality, and aridity index (van Wart et al. 2013). The framework evaluates all CZs that account for > 5% of total national harvested area for each crop (either irrigated or rainfed water regime). Within each CZ, buffer zones of 100-km radius (called “sites” in main text) were created around existing weather stations where measured weather data were retrieved (Supplementary Figure S1). Each buffer was “clipped” by CZs borders so that temperature and rainfall regimes were relatively uniform within buffers. For each crop-water regime, buffers were selected sequentially starting from the buffer with largest harvested crop area, only including buffers that account for > 1% of national crop harvested area and minimizing overlap (< 20%) among adjacent buffers, until approximately half the total national harvested area was covered for the target crop. Crop area distribution maps of maize and rice around 2005 (average for 2004–2006), disaggregated by water regime, were retrieved from the International Food Policy Research Institute (IFPRI—MAPSPAM database 2016, http://www.mapspam.info). MAPSPAM provides 10 × 10 km grid-cell resolution maps of harvested area for each of the major food crops. In a few cases (14%) there were no weather stations in areas where new cropland was established. Additional buffers were created in selected producing regions without MAPSPAM data or where there were no weather stations. In the last case, we used secondary gridded weather data from the NASA-POWER database (POWER 2017, http://power.larc.nasa.gov). A total of 50, 55, and 16 buffers were created for irrigated rice in China, rainfed or irrigated rice in Indonesia, and rainfed maize in Nigeria, respectively.

Within each buffer, dominant soil types and crop management data were obtained from the GYGA database to portray the dominant cropping system(s) used for simulation of annual yield potential. In summary, crop management, soil, and climate factors governing yield potential, as well as sub-national data on current farm yields reported by government agencies, were populated at the buffer level using observed data to the extent possible. Upscaled estimates of current yields and yield potential at CZ scales were based on aggregation of crop area-weighted values of all buffer zones within each CZ. A detailed description of the GYGA spatial upscaling methodology can be found elsewhere (Grassini et al. 2015; van Bussel et al. 2015; http://www.yieldgap.org).

In selection of crop models, we gave preference to models that have been rigorously evaluated for their ability to reproduce yields in absence of nutrient limitations and biotic stress. We used the ORYZA v3 crop model (Li et al. 2017) to simulate yield potential of irrigated rice in China and lowland rainfed and irrigated rice in Indonesia, and the Hybrid Maize model (Yang et al. 2017) for simulating yield potential of rainfed maize in Nigeria. Both models simulate crop growth and development on a daily time step. Growth rates are determined by simulation of both CO_2_ assimilation and respiration with partitioning coefficients to different organs dependent upon developmental stage. Cultivar-specific coefficients for dominant rice varieties in China and Indonesia were obtained from Agustiani et al. 2018 and Deng et al. 2019, respectively. Hybrid Maize requires a single genotype-specific input parameter: growing degree days from crop emergence to physiological maturity. All other parameters governing photosynthesis, respiration, leaf area expansion, light interception, biomass partitioning, and grain filling are considered to be stable across modern maize hybrids. In all cases, simulations of yield potential assumed absence of insect pests, weeds, and diseases and no nutrient limitations. To estimate rainfed yield potential, both models account for timing and amount of rainfall as well as soil properties influencing crop-water availability. Both ORYZA v3 and Hybrid Maize models have been widely evaluated on their ability to reproduce measured crop phenology, biomass, yield, and other agronomic traits in well-managed experiments (van Ittersum et al. 2016; Agustiani et al. 2018; Deng et al. 2019). Simulations of yield potential were based on 10–15 years of weather data and dominant crop sequences at each site. In the case of rainfed crops, yield potential was simulated separately for the 2–3 dominant soil types within the buffer where the climate station was located. For each site, yield potential was estimated by averaging the yield potential simulated for the different combinations of crop sequence and soil type after weighting them for their relative share of rice or maize harvested area within each buffer. In the case of rainfed crops, initial soil water was estimated by initializing the model’s soil water routine at the beginning of the preceding fallow period or using a fixed soil water content at sowing as determined by expert opinion. Current yields were obtained from official statistics at the lowest administrative level at which they are available within each buffer, for the most recent five years. Using a longer time period would bias estimation of current yields due to influence of technology adoption trends (van Ittersum et al. 2013). Details on data sources to estimate yield potential in each country and data sources can be found in Supplementary Table S3 and elsewhere (van Ittersum et al. 2016; Agustiani et al. 2018; Deng et al. 2019).

### Impact of land conversion on production of key staple crops

Three countries undergoing rapid urbanization during the last few decades were selected as case studies (http://www.oecd.org). As a first step, we evaluated the association between the magnitude of population growth in major cities (United Nations 2018) and changes in total crop area of seven primary staple crops (rice, maize, wheat, soybean, barley, sorghum, and cassava) from 2000 to 2010 in China. We used crop area distribution in 2000 (average for 1999–2001) and 2010 (2009–2011) (IFPRI 2019a) from MAPSPAM to estimate net change in crop area for that 10-year period in individual spatial grids of roughly 30 000 km^2^. Ten years provides a timeframe that is long enough to assess changes in cropland, while using 3-year averages helps reduce the influence of short-term changes in cropland area due to fluctuation in market prices or unusual weather events like drought or floods. Similarly, we used MAPSPAM to determine the expansion or contraction in crop area within each buffer and CZ, for specific staple crops in Nigeria (maize), China (rice) and Indonesia (rice) between 2000 and 2010. In Nigeria, maize is grown under rainfed conditions, which means crop growth depends on stored soil water at sowing and in-season rainfall to meet its water requirements. In China, nearly all rice is irrigated, while both irrigated and lowland rainfed rice are grown in Indonesia.

Current yields and yield potential, as well as crop intensity and yield stability, in buffers experiencing large cropland conversion of rice or maize were compared with those at buffers where cropland is currently expanding (Fig. 2). We estimated yields on an annual basis to account for the higher crop intensity in those regions where two or even three crops were produced each year on the same piece of land (rice in Indonesia and southern China). Then, for each country, we calculated the average annual yield (either current or potential) in CZs with expanded or contracted crop-specific area, weighted by the crop area net balance in buffers within each CZ (2000–2010). National average yield in contracting or expanding areas were calculated separately based on yields at the CZ level according to the crop-specific area share of each CZ. For each country, the ratio between the yield in CZs where crop area is contracting and the yield in CZs where cropland is expanding was estimated as follows:

1 $${\text{National}}\;{\text{yield}}\;{\text{ratio}} = \frac{{{\text{Weighted}}\;{\text{yield}}\;{\text{in}}\;{\text{contracting}}\;{\text{CZ}}}}{{{\text{Weighted}}\;{\text{yield}}\;{\text{in}}\;{\text{expanding}}\;{\text{CZ}}}}.$$

A yield ratio greater than one means that the new crop-specific area is less productive than the one being converted to other uses and, therefore, proportionally more land is needed to compensate for each hectare lost. For comparison, yield ratios were estimated separately based on either current yields (Supplementary Table S2) or potential yields as reported in the main text. In this study, yields are reported at 15.5% and 14% seed moisture for maize and rice, respectively, which correspond to the commercial yield reporting standards for these crops.

## Results

**Fig. 1:**
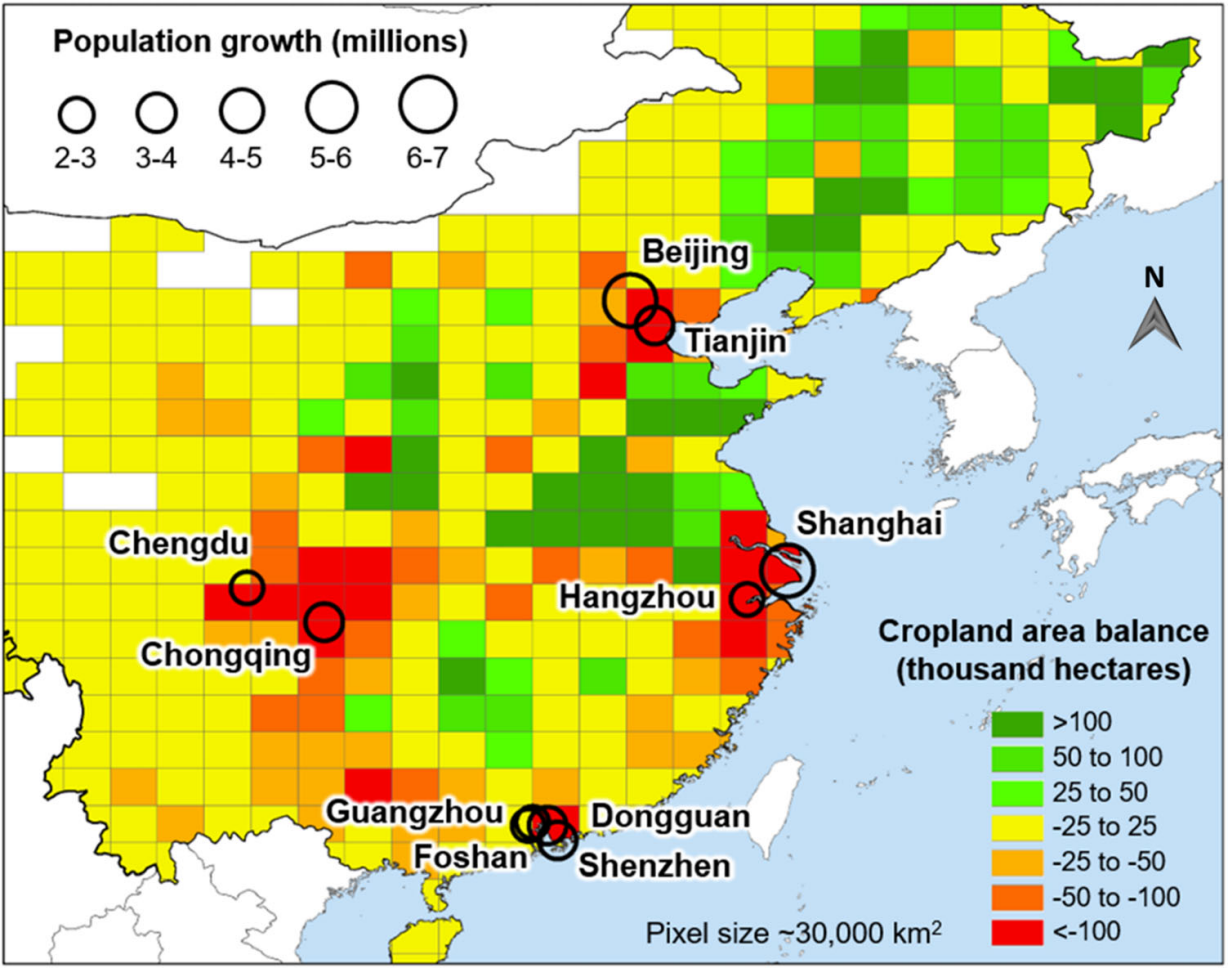
Changes in cropland area for seven major staple food crops (rice, maize, wheat, soybean, barley, sorghum, and cassava) (IFPRI 2019b; http://www.mapspam.info) and changes in population of major cities from 2000 to 2010 (United Nations 2018). Labeled cities correspond to urban centers with population growth larger than 2 million inhabitants in the 2000–2010 period

Our study shows that population growth has been a key driver of cropland change (Fig. 1). For example, in China, total production area of seven major staple food crops decreased substantially in regions surrounding the most rapidly growing cities during the 2000–2010 period. In contrast, cropland expansion occurred in central and northeastern China where urban population growth was much slower. Given the magnitude and extent of land-use change, assessing yield differences of both converted and new cropland is necessary to determine the impact of these changes on national crop production potential.

**Fig. 2:**
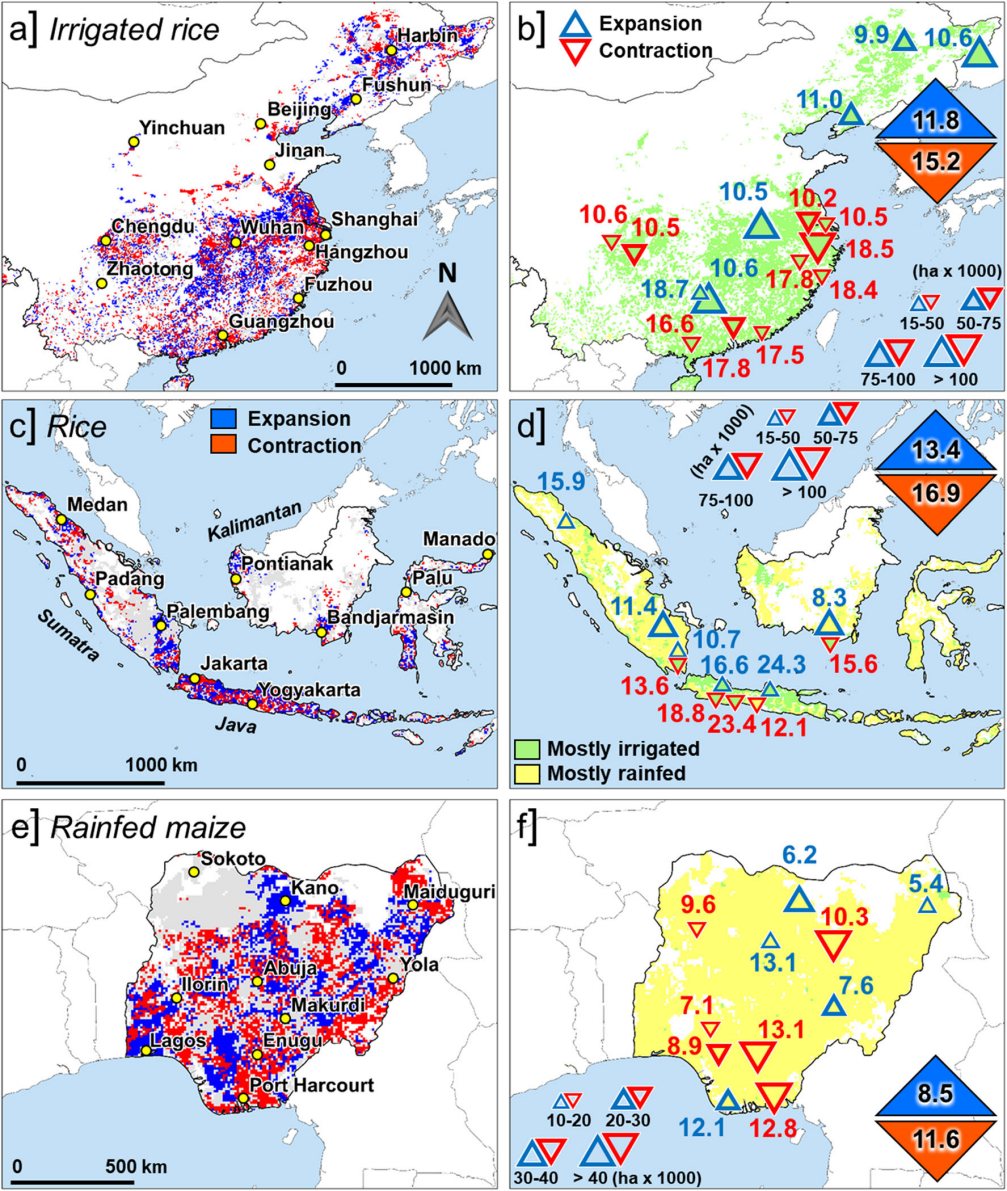
Left panels: net change in cropland area (2000–2010) in **a** China (irrigated rice), **c** Indonesia (rainfed and irrigated rice), and **e** Nigeria (rainfed maize) (IFPRI 2019c; http://www.mapspam.info). Colored areas are those with at least 50 hectares of cropland per pixel (each approximately 10 000 ha). Regions where crop area is contracting are shown in red, while regions where crop area is expanding are shown in blue (> 250 ha change per pixel in both cases). Right panels **b**, **d**, **f**: annual yield potential (t ha^−1^) at sites with significant change in net area balance (greater than 15 000 and 10 000 hectares for rice and maize in the period from 2000 to 2010, respectively; blue triangles: positive net change, red inverted triangles: negative net change). Yields at sites with no significant change in net area (smaller than 15 000 or 10 000 hectares for rice and maize, respectively) are not shown. Weighted national yield averages (insets in right panels) were calculated using GYGA aggregation procedures based on a climate zone spatial framework (van Bussel et al. 2015). A number of administrative capitals are shown as a reference in left panels

One reason for differences in total grain production between converted and new cropland is cropping intensity (i.e., number of crops grown each year on the same piece of land). In China, irrigated rice area has been decreasing in regions surrounding mega cities such as Shanghai (current population: 27 M (United Nations 2018)), Guangzhou (13 M), and Hangzhou (8 M) where warm climate and long growing season allow production of two rice crops per year on the same field (called double cropping). Expansion of irrigated rice production occurred mostly in central and northeastern regions where only a single rice crop can be grown each year given a cooler climate and shorter frost-free period (Fig. 2a). National average annual yield potential of converted rice land was 15.2 t ha^−1^ compared with 11.8 t ha^−1^ for newly established rice land (Fig. 2b). While yield potential for a single crop is highest in central and northern provinces, total annual production potential per hectare is about 70% greater in south and southeast of China due to annual double cropping. Taking into account all areas undergoing cropland conversion or expansion, the area-weighted national yield ratio in China is 1.3 (Table 1), which means that, on average at national scale, proportionally more cropland (1.3×) is required to replace the productive potential of one hectare of rice land lost. Not accounting for differences in cropping intensity would lead to the (wrong) conclusion that new crop area in China has higher rice yield potential than crop area lost. Yield stability on new and converted cropland, as quantified by the inter-annual coefficient of variation (CV), is similar in both cases because rice is produced with irrigation, which avoids yield losses from drought and greatly increases yield stability compared to rainfed crop production.

**Table 1:** Yield ratios estimated by comparing the annual yield potential of areas converted to other uses versus new areas brought into crop production during the 2000–2010 period. Crop intensity refers to the number of crops of rice (China and Indonesia) or maize (Nigeria) grown each year on the same piece of land

Parameter	China		Indonesia		Nigeria	
	Irrigated rice		Rice^a^		Rainfed maize	
	Converted	New	Converted	New	Converted	New
Crop intensity (crops year^−1^)	1.7	1.2	1.7	1.4	1	1
Irrigation proportion (%)	100	100	94	20	Nil	Nil
Annual yield potential (t ha^−1^)	15.2	11.8	16.9	13.4	11.6	8.5
Yield ratio	1.3	–	1.3	–	1.4	–
Yield stability (CV in %)	8	8	4	3	27	51

Yield stability is estimated by the inter-annual coefficient of variation in annual yield potential

^a^Includes irrigated and lowland rainfed rice

A similar situation occurs in Indonesia where highly productive irrigated rice area in West and Central Java, the island with fastest population growth (+ 122 inhabitants km^−2^ year^−1^ in 2000–2010; http://www.bps.go.id), has been converted to other uses (Mulyani et al. 2016), while rice area expanded mostly into more marginal agricultural regions, with slower population growth, such as in South Sumatra (+ 14) and South Kalimantan (+ 17) (Fig. 2c). Total annual yield potential is about two-fold greater in irrigated double (or even triple) rice systems in West and Central Java compared with those marginal regions, where single-crop tidal and flood-prone rice systems are dominant (Fig. 2d). In these harsher environments, rice cultivation depends on ocean tides and rainfall, which typically allow growing only one rice crop per year, which, in turn, has lower yield potential due to exposure to both flooding and drought stress. Considering all land conversion and expansion throughout the country, national average yield ratio in converted *versus* new rice land in Indonesia is 1.3 (Table 1).

In Nigeria, the greatest reduction in rainfed maize area occurred in southern coastal regions with humid tropical climate around Port Harcourt (+ 0.7 M population increase, 2000–2010). Most new maize area came from northward expansion into the more sparsely populated Guinea Savanna region, which has lower annual rainfall and greater year-to-year variation in rainfall amounts (Fig. 2e). As a consequence, rainfed yield potential of new maize land is considerably lower and much less stable than the converted land it replaced, with a national average yield ratio of 1.4 (Fig. 2f; Table 1). In contrast to Indonesia and China, farmers in most of Sub-Saharan Africa lack adequate access to inputs and extension services. As a result, the difference in potential productivity between new *versus* converted land reported here is not captured when the analysis is based on the very low current yields attained by maize farmers throughout the country (1.8 t ha^−1^) (Supplementary Table S2).

Sub-national estimates of annual yield potential for new and converted cropland show enormous variation due to endowments of climate and soil. For example, across rice producing regions in China, total annual yield potential ranges from 10 to 19 t ha^−1^ in both new and converted croplands (Fig. 2). Similarly, wide ranges of annual production potential can be observed across rice and maize producing areas in Indonesia and Nigeria, respectively. Hence, national average yield ratios based on area-weighted estimates of annual yield potential, as given in Table 1, hide enormous variation in sub-national estimates of annual production potential (Fig. 2). As a result, using a fixed ratio to estimate the impact of land conversion on crop production at national level can give misleading input to inform national agricultural and land-use policies, including prioritization of investments in agricultural research and development.

## Discussion

Accuracy of estimated yield potential differences between converted and new cropland is sensitive to data quality, precision of cropland distribution maps, the spatial scale at which data are analyzed and aggregated, and reliability of crop yield potential simulations. We have confidence in the spatial framework used for upscaling results from sub-national to national scale because it has proven to be robust in estimating yield potential at sub-national to national scales for a number of crops and countries across a wide range of soils and climates (Rattalino Edreira et al. 2021; van Wart et al. 2013; Hochman et al. 2016; Deng et al. 2019). Likewise, crop simulation models used to evaluate yield potential have been widely validated in China, Southeast Asia, and Sub-Saharan Africa (van Ittersum et al. 2016; Agustiani et al. 2018; Deng et al. 2019). While we attempted to use the best available sub-national data sources for cropland distribution, cropping systems, climate, and soil properties as described by Grassini et al. (2015), data quality is always a concern for long-term weather records and soil properties, which are input to the simulation models and, thus, may be a source of uncertainty (http://www.yieldgap.org). Similarly, crop models may not account for all possible factors limiting crop production. For example, currently available rice simulation models do not account for the negative effect of alternate cycles of drought and submergence, which are frequent in tidal and flood-prone systems of the new Indonesian rice production areas but less common in regions with irrigated production (Mackill et al. 1996). Similarly, the best available rice models have limited ability to simulate the effects of cold sterility (van Oort et al. 2015), which may be important for estimating yield potential in high-latitude temperate environments as found in northeastern China where rice production area is expanding. Our estimated yield ratios can also change in the future due to climate change, which will alter yield potential in most regions. For example, some low-lying areas where rice is currently cultivated will be subject to increasing problems of floods and land degradation due to saltwater intrusion and subsidence (Rondhi et al. 2019). Inclusion of these factors in simulating yield potential would tend to increase estimated yield ratios between converted and newly developed croplands as found in this study. Finally, having more than two time points and a longer timeframe would have been desirable to better account for the influence of policies and interventions within the agricultural sector on cropland changes and would avoid confounding effects of changes in crop area due to fluctuation in grain prices. However, this is not possible given current data availability on crop-specific area distribution.

Current trends indicate that increases in the supply of staple food crops rely more on crop area expansion than on the rise in yields, which reverses trends of previous decades when crop yield increases were more prominent (Cassman and Grassini 2020). Reliance on conversion of new land to meet increasing food demand is amplified by loss of existing farmland to urbanization. Hence, estimation of the impact from these trends on food production capacity provides critical input to development of agricultural and land-use policies at national and global scales to achieve appropriate balance between food security and environmental goals. Using locally collected crop management, soil, and weather data and robust simulations of crop yield potential and yield stability, we found that average national yield ratios between converted and newly developed croplands range from 1.3 for rice in China and Indonesia to 1.4 for maize in Nigeria. Despite relatively little variation in these national averages, there was enormous variation in yield potential of both converted and new land at sub-national scales in all three countries. Hence, average ratios should be used with caution as input to strategic national land-use plans. Similarly, use of current yields (d’Amour et al. 2017), rather than yield potential, underestimates the impact on productivity by a large margin when average farm yields in both converted and new land are limited by lack of inputs and technologies to overcome nutrient deficiencies, weeds, and pests, which is the case for maize in Nigeria. In addition, year-to-year variation in Nigerian rainfed maize yield potential is two-fold larger on new rainfed maize land than on maize area lost (Table 1), which means food production on new land is much less reliable than on converted land. Similar assessments are possible for other countries that have sub-national data on changes in population (United Nations 2018), crop production area (IFPRI 2019a; http://www.mapspam.info), and crop production systems, soils, and climate (http://www.yieldgap.org).

Conversion of key staple cropland for urban use can be penny wise when substantial profits accrue from such land development. But these conversions can also be pound foolish for several reasons when new cropland has substantially lower yield potential, less yield stability, or both. First, a yield ratio greater than one increases pressure to further expand cropland area to meet food demand through conversion of rainforests and grasslands at the expense of biodiversity and other ecosystem services provided by natural habitat. Second, deforestation and conversion to agricultural land use accounts for 17% of global greenhouse gas emissions contributing to climate forcing (Barker et al. 2007). Third, urbanization of croplands with irrigation infrastructure and cropland expansion toward rainfed areas with great year-to-year variation in rainfall make food production more vulnerable to expected climate change, which includes more erratic and extreme rain events (Mereu et al. 2015; Rondhi et al. 2019). Fourth, in rainfed systems, reduced yield stability makes it riskier to invest in fertilizer and other inputs to raise yields in new production areas, which in turn contributes to slower rates of increase in crop yields (Grassini et al. 2015, Sadras et al. 2019). And fifth, while we assessed impact based solely on differences in annual crop yield potential, the overall cost would be higher if one also considers the greater production costs (fertilizer, labor, transportation) and required investments in infrastructure (roads, canals, drainage systems) associated with establishing crop production in remote areas where expansion typically occurs. National demand for rice will increase substantially in China and Indonesia (Agus et al. 2019; Deng et al. 2019) and for maize in Nigeria (van Ittersum et al. 2016). Hence continuation of current cropland trends, with net loss and gain in most and less productive regions, respectively, will put further pressure on closing yield gaps and/or increased reliance on grain imports to meet the domestic demand.

## Conclusions

We conclude that in countries where yield ratios between converted and new land are large, as found in this study, there is strong justification for land-use policies that seek to conserve prime farmland dedicated to cultivation of key staple crops at the periphery of urban areas. This must be supported by agricultural development and land-use policies seeking to accelerate yield gains on existing farmland through sustainable intensification while also ensuring conservation of natural ecosystems (Garnett et al. 2013; d’Amour et al. 2017; Cassman and Grassini 2020). Continuing current land-use trajectories undermines progress toward the tripartite goals of food security, conservation of natural resources, and addressing the threat of climate change.

## Supplementary Information

Below is the link to the electronic supplementary material.

Supplementary file1 (PDF 1157 kb)

## Data Availability

The datasets generated and/or analyzed during the current study are available from the Global Yield Gap Atlas website (www.yieldgap.org). Crop area layers are available at SPAM website (http://www.mapspam.info).
