# Supplementary material for: Impact of urbanization trends on production of key staple crops
Source: Ambio. 2021 Nov 29;51(5):1158–67. doi: 10.1007/s13280-021-01674-z (PMC8931132; doi:10.1007/s13280-021-01674-z)
Supplement: Supplementary file 1 — Supplementary file1 (PDF 1157 kb) [file 13280_2021_1674_MOESM1_ESM.pdf]

Ambio

Supplementary Information
This supplementary information has not been peer reviewed.

Title: **Impact of urbanization trends on production of key staple crops**

Supporting information

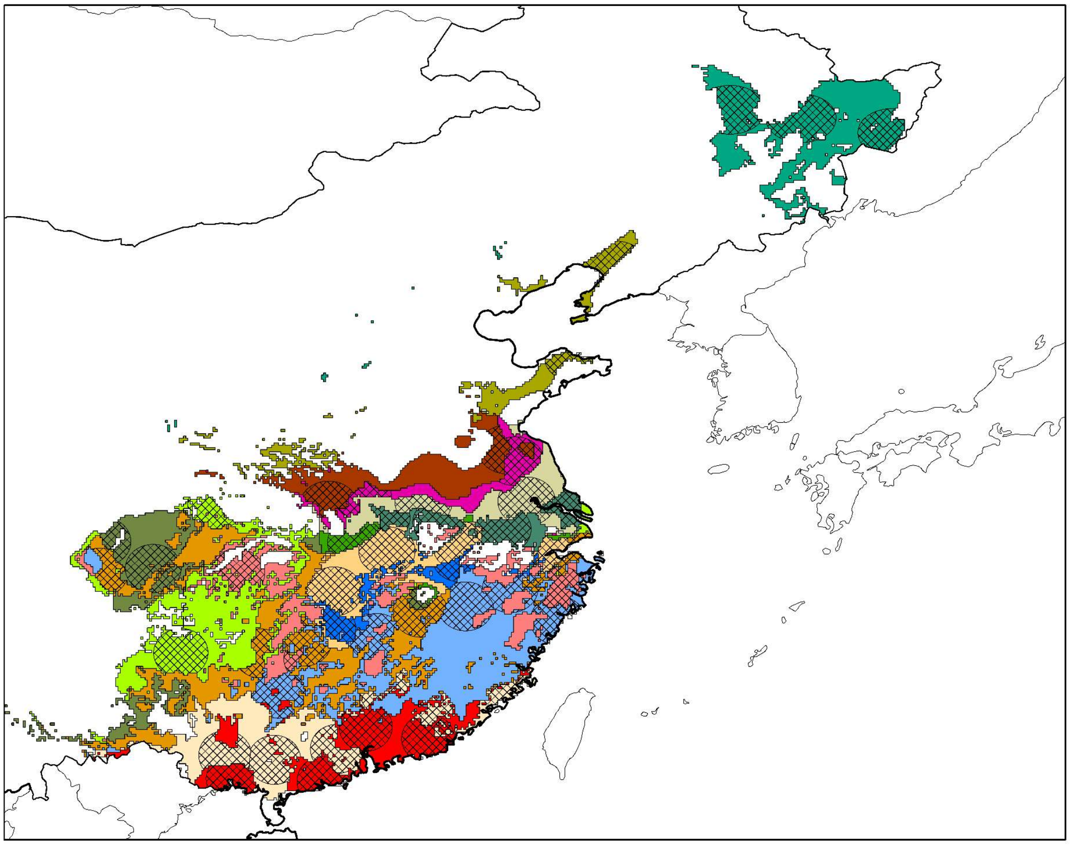

Supplementary Figure S1. *Colored areas indicate climate zones with >5% of national irrigated rice cropping area in China. Hatched areas correspond to 100-km radius buffers zones surrounding weather stations, clipped by climate zones borders.*

Supplementary Table S1. *Yield and land cover data sources and yield ratio estimation in previous studies as compared with this research article.*

Reference	Yield source	Land use source	Spatial framework	Yield ratio estimation
Hertel et al., 2010	None.	Global Trade Analysis Project (GTAP).	None.	Assumed as constant: 1.5 hectares of new cropland (anywhere) are needed to compensate each hectare of converted cropland (anywhere).
Taheripour et al., 2012	Yield potential estimated based on annual primary net productivity using satellite images. Discriminates between C3 and C4 crop species but is not crop specific.	Global Trade Analysis Project (GTAP).	Global Agro-Ecological Zones.	Estimated as the ratio between estimated yield potential in agricultural *versus* non-agricultural lands that may be converted into croplands.
d'Amour et al., 2017	Current yield and harvested area data from the atlas of global crop areas and yields in the year 2000, which is constructed from national statistics at district level, validated with FAOSTAT yield data around year 2000. Yields are then transformed to calories weighted by harvested area of every crop so results are not crop-specific.	Prospects on *land use*, global forecast of urban expansion by 2030.	None.	Gridded yield maps were transformed into calories considering harvested area of every crop on each pixel. Results are not crop-specific.<br><br>Substitution cost is then calculated as the ratio between calories produced in urbanized croplands *versus* the national/regional average.
van Vliet, 2019	Current yields from the atlas of global crop areas and yields in the year 2000. Database constructed based on national statistics at district level, validated with FAOSTAT yield data around year 2000.<br><br>Yields potential from Global Agro-Ecological Zones (GAEZ) project, which uses generic models for simulations and gridded data sources. Not crop-specific.	European Space Agency Climate Change Initiative (ESA CCI) Land Cover data.	Direct upscaling of yields from grid to country and subcontinental regions.	Gridded yield maps were transformed into calories considering harvested area of every crop on each pixel. Results are not crop-specific.<br><br>Calculated as the average annual productivity of cropland converted into urban land and the average productivity of new cropland areas that may be used to compensate for the loss in crop production.
This article.	Current yields calculated from national statistics, at district level, using the most recent five-year period available.<br><br>Yield potential simulated using site-specific data on crop management, weather, and soils with locally-calibrated crop models	Spatial Production Allocation Model (SPAM) by HarvestChoice	Global Yield Gap Atlas	Calculated as the ratio between annual yields of specific crops in sites with net area expansion versus sites with net area contraction in 2000-2010.

Supplementary Table S2. *Yield ratio estimated by comparing the annual current yield of the cropland converted to other uses versus the new area brought into crop production. Crop intensity refers to the number of crops of rice or maize grown each year on the same piece of land. Yield stability is estimated by the coefficient of variation in annual current yields. Yield gaps are expressed relative to the yield potential presented in Table 1.*

	**China** Irrigated rice		**Indonesia** Rice[†]		**Nigeria** Rainfed maize	
	Converted	New	Converted	New	Converted	New
Average crop intensity (crops y^{-1})	1.7	1.2	1.7	1.4	1	1
Irrigation proportion (%)	100	100	94	20	0	0
Annual current yield (t ha^{-1})	10.1	8.5	10.2	7.1	1.8	1.8
Yield gap (as % of yield potential)	34	28	40	47	84	79
Yield ratio	1.2	-	1.4	-	1.0	-
Yield stability (CV in %)	N.A.	N.A.	3	4	10	20

[†]Includes irrigated and lowland rainfed rice. N.A.: not available

Supplementary Table S3. *Crop, water regime, crop model used for yield potential assessment, number of years simulated, and data sources of weather, soil, crop management, current yields, and cropland distribution at each country.*

**Country**	**China**	**Indonesia**	**Nigeria**
Crop	Rice	Rice	Maize
Water regime	Irrigated	Rainfed and irrigated	Rainfed
Crop model for yield potential assessment	Oryza v3	Oryza v3	Hybrid-Maize
Simulated years	11 (2004-2014)	15 (2001-2015)	14 (1999-2012)
Weather data	National Meteorological Information Center of the China Meteorological Administration	Indonesian Agency for Meteorological, Climatological and Geophysics	Nigerian Meteorological Agency
Soil water holding capacity	Not needed for irrigated crops	Indonesian Center for Agricultural Land Resources Research and Development	AfSIS-GYGA functional soil information for Sub-Saharan Africa
Crop management	Local experimental data and publications	Researchers and extension workers from the Assessment Institute for Agriculture Technology	Local country agronomists
Current yield	National and provincial statistical bureaus revised by Deng et al. (2019)	Badan Pusat Statistik	National bureau of Statistics of Nigeria
Cropland distribution	Spatial Production Allocation Model	Spatial Production Allocation Model	Spatial Production Allocation Model
More details	Deng *et al*. (2019)	Agus *et al*. (2019)	van Ittersum *et al*. (2016)

Additional references from Supplementary Information

Hertel, T.W., A.A. Golub, A.D. Jones, M. O'Hare, R.J. Plevin, and D.M. Kammen. 2010. Effects of US maize ethanol on global land use and greenhouse gas emissions: Estimating market-mediated responses. *BioScience* 60: 223–231.

Taheripour, F., Q. Zhuang, W.E. Tyner, and X. Lu. 2012. Biofuels, cropland expansion, and the extensive margin. *Energy Sustainability and Society* 2: 1–11.
